# A Pilot Study of Mifepristone in Combat-Related PTSD

**DOI:** 10.1155/2012/393251

**Published:** 2012-04-24

**Authors:** Julia A. Golier, Kimberly Caramanica, Rebecca DeMaria, Rachel Yehuda

**Affiliations:** ^1^Department of Psychiatry, James J. Peters VA Medical Center, Bronx, NY 10468, USA; ^2^Department of Psychiatry, Mount Sinai School of Medicine, New York, NY 10029-6574, USA

## Abstract

*Background*. We obtained pilot data to examine the clinical and neuroendocrine effects of short-term mifepristone treatment in male veterans with PTSD. *Methods*. Eight male veterans with military-related PTSD completed a randomized, double-blind trial of one week of treatment with mifepristone (600 mg/day) or placebo. The primary clinical outcome measures were improvement in PTSD symptoms and dichotomously defined clinical responder status as measured by the CAPS at one-month follow-up. Additional outcome measures included self-reported measures of PTSD symptom severity, CAPS-2 symptom subscale scores, and morning plasma cortisol and ACTH levels. *Results*. Mifepristone was associated with significant improvements in total CAPS-2 score. At one-month follow-up, all four veterans in the mifepristone group and one of four veterans in the placebo group achieved clinical response; three of four veterans in the mifepristone group and one of four veterans in the mifepristone group remitted. Mifepristone treatment was associated with acute increases in cortisol and ACTH levels and decreases in cytosolic glucocorticoid receptor number in lymphocytes. *Conclusions*. Further controlled trials of the effects of mifepristone and their durability are indicated in PTSD. If effective, a short-term pharmacological treatment in PTSD could have myriad uses.

## 1. Introduction

 Within the last decade, two selective serotonin reuptake inhibitors (SSRIs), sertraline and paroxetine, have been approved by the FDA for the treatment of posttraumatic stress disorder (PTSD). In studies of civilian trauma survivors, each has been superior to placebo in achieving clinical response and reducing core PTSD symptoms, which represents a significant advance. However, their use as PTSD monotherapy has significant limitations. Even in the most favorable studies, some key symptoms are resistant to treatment (e.g., sleep disturbance), remission is uncommon, and continuous treatment is often necessary to prevent relapse. Additionally, SSRIs have been shown to be largely ineffective in combat veterans with PTSD. There is a need to develop better pharmacological treatments that specifically target PTSD symptoms and/or pathophysiology. Given the abundant evidence for hypothalamic-pituitary adrenal (HPA) axis dysregulation in PTSD, we performed a preliminary study to examine whether mifepristone—which recalibrates the HPA axis through peripheral and central mechanisms—could be of therapeutic benefit.

Mifepristone is a selective antagonist of glucocorticoid receptors (GRs) that induces increases in cortisol and adrenocorticotropin hormone (ACTH) levels through blockade of cortisol's feedback inhibition of the HPA axis. At high doses, mifepristone has central effects [1, 2]. Mifepristone and its metabolites appear to retard the rate at which brain cortisol is expelled via the glycogen pump at the blood-brain barrier, effectively increasing cortisol levels in the brain [1, 2]. This acute increase in cortisol leads to an upregulation of hippocampal mineralocorticoid receptors (MRs); re-regulation of the balance of MR and GR receptors is thought to play a role in the recalibration of the HPA axis [3, 4]. Since PTSD is associated with glucocorticoid alterations including enhanced glucocorticoid sensitivity [5] and elevated corticotropin releasing factor (CRF) levels [6, 7], short-term administration of mifepristone may reverse some of the stress-related neurobiological changes associated with this disorder and restore homeostasis to this system.

Preliminary data suggest that short-term administration of high doses of mifepristone can have beneficial effects in other neuropsychiatric conditions [3, 8–10], but to date mifepristone has not been studied in PTSD. In this randomized, double-blind, placebo-controlled pilot study, we sought to collect preliminary data on the clinical and neuroendocrine effects of short-term (7 day) treatment with high-dose (600 mg/day) mifepristone in veterans with PTSD in order to guide further investigation of its clinical benefits and mechanism of action in PTSD.

## 2. Materials and Methods

Male veteran outpatients who met DSM-IV criteria for chronic military-related PTSD and who were without a history of adrenal insufficiency (or morning plasma cortisol <5 mcg/dL), diabetes mellitus or endocrinopathy, a history of moderate to severe traumatic brain injury, stroke, or other neurological illness, a history of schizophrenia, schizoaffective disorder, or bipolar disorder, current suicidal ideation, or a known allergy to mifepristone were eligible to participate. Veterans who were taking oral corticosteroids, receiving specialized trauma-focused psychotherapy, or who were unwilling to use effective contraceptive methods during the study were excluded. Veterans receiving psychotropic medications for PTSD were eligible to participate if they had been stabilized at a therapeutic dose for a minimum of six weeks prior to randomization. The protocol was approved by the Institutional Review Board of the James J. Peters VA Medical Center. Written informed consent and HIPAA authorization were obtained from all participants prior to the initiation of any study procedures.

 Participants underwent a complete diagnostic assessment, including a medical history, physical examination, laboratory testing, and administration of the Clinician Administered PTSD Scale (CAPS) and the Structured Clinical Interview for DSM-IV (SCID). Questionnaires were administered to assess combat exposure severity and characteristics of deployment (combat exposure questionnaire (CEQ)), childhood trauma (childhood trauma questionnaire (CTQ)), and symptoms of depression (beck depression inventory (BDI)) and posttraumatic stress (PTSD checklist (PCL)).

 Eligible veterans were randomized to receive mifepristone (600 mg/day) or matched placebo for one week (7 days), to be taken at bedtime. The randomization log was maintained by the VA pharmacy, and both the participant and research staff were blinded to the treatment assignment. Clinical assessments were performed at baseline, treatment endpoint, and four weeks after drug discontinuation. Vital signs, adverse events, and concomitant medications were obtained at every visit to monitor safety.

 The primary clinical outcome measures were severity of PTSD symptoms, as measured by the total severity score of the CAPS-2, and dichotomously defined clinical responder status. The liberal responder criterion, defined as a 12-point or greater reduction in total CAPS-2 score, reflects reliable clinical changes in veterans with PTSD. The more conservative criterion, a 30% or greater reduction in total CAPS-2 score, is a frequently used definition in PTSD clinical trials. Secondary clinical outcome measures included self-reported symptoms as measured by the PCL and the BDI, and the three PTSD symptom subscale scores from the CAPS-2 (intrusive symptoms, avoidance symptoms, and hyperarousal symptoms). Dichotomous remitter status, defined as no longer meeting diagnostic criteria for PTSD at four-week follow-up, was also explored.

Morning plasma cortisol and ACTH levels and GR binding per cell were measured as indicators of the magnitude of mifepristone's effects on negative feedback inhibition. The lysozyme IC_50-DEX_ was used to measure mifepristone's antagonism of peripheral GR sensitivity [5].

 Measures of central tendency and variability (mean and SD) were calculated at treatment baseline, endpoint, and four-week follow-up for all continuous primary and secondary outcome measures. Baseline comparisons of group differences were conducted using independent samples *t*-tests for continuous variables and chi-square analysis for categorical variables. Changes within groups for the primary and secondary outcome measures were analyzed using a series of paired samples *t*-tests, pairing initial rating and endpoint or four-week follow-up. For between groups-analysis, changes in primary and secondary outcome measures were analyzed using independent samples *t*-tests. The Mann-Whitney *U* test, which compares the sum of the ranks and is less likely to be influenced by outliers, was used to assess whether findings from the independent samples *t*-tests remained significant when nonparametric methods were employed. The dichotomous clinical response and remitter status variables were analyzed using chi-square analysis. All tests were two sided with statistical significance set at *P* < 0.05.

## 3. Results

 Thirteen veterans were enrolled in the clinical trial; three were lost to follow-up or found to be ineligible, one withdrew prior to randomization, nine were randomized, and eight completed study procedures. For completers (*n* = 8), the mean (SD) age was 48.88 (12.68) years (range 26–63 years). With respect to race and ethnicity, 12.5% were Caucasian, 75% were African American, and 12.5% chose not to identify their race; 25% were Hispanic. The mean level of education was 13.5 (2.39) years. Four participants served in the Vietnam or post-Vietnam era, and four participants were Operation Iraqi Freedom/Operation Enduring Freedom (OIF/OEF) veterans. The mean score on the CEQ at screening was 55.88 (12.26), indicating moderate exposure to combat stress. As shown in Table 1, there were no significant differences between groups for multiple clinical variables at baseline.

 Based on the clinical response definition of a 12-point or greater decrease in total CAPS-2 score from initial evaluation, three of four veterans in the mifepristone group and two of four veterans in the placebo group achieved clinical response at treatment endpoint (*P* = 0.465). At one-month follow-up, all four veterans in the mifepristone group and one of four veterans in the placebo group achieved clinical response (*P* = 0.028). Similar improvements were observed using the clinical response definition of a 30% change in total CAPS-2 score; three of four veterans in the mifepristone group achieved clinical response at treatment endpoint compared to one of four in the placebo group (*P* = 0.157). At one-month follow-up, two of four veterans in the mifepristone group and no veterans in the placebo group maintained clinical response (*P* = 0.102). With respect to remitter status, one of four veterans in the placebo group and three of four veterans in the mifepristone group no longer met diagnostic criteria for PTSD at four-week follow-up (*P* = 0.157).

Clinical data are shown in Table 1 for participants treated with mifepristone or placebo. Between groups analysis showed that the mifepristone group had significantly greater improvement in total CAPS-2 score (*P* = 0.047) at one-month follow-up when compared to the placebo group. Using the Mann-Whitney *U* test, our findings were similar to those obtained using parametric methods, with significant improvement in total CAPS score (*P* = 0.021) across groups from screening to four-week follow-up. The mifepristone group also had a significantly greater improvement in CAPS-2 avoidance symptom scores at four-week follow-up when analyzed with both parametric (*P* = 0.027) and nonparametric (*P* = 0.028) methods. Within-groups analysis showed a significant decrease in total CAPS-2 score from initial evaluation to four-week follow-up (*P* = 0.039) in the mifepristone group; this included significant decreases in intrusive symptom score (*P* = 0.001) and avoidance symptom score (*P* = 0.008). There were no significant changes in total CAPS-2 score or CAPS-2 symptom subscale scores within the placebo group. The mifepristone group also had a significant decrease in PCL score at four-week follow-up (*P* = 0.036). There were no significant differences between groups for improvement in PCL or BDI scores.

 Neuroendocrine outcomes are also presented in Table 1; significant changes in plasma cortisol (*P* = 0.007), ACTH (*P* = 0.009), and GR binding (*P* = 0.041) from baseline to treatment endpoint were observed within the mifepristone group. There were no significant biological changes in the placebo group, and neither group showed significant changes in lysozyme IC_50-DEX_. Between-groups analysis revealed significant differences in changes in cortisol (*P* = 0.001), ACTH (*P* = 0.009), and GR binding (*P* = 0.003) from baseline to treatment endpoint. Nonparametric tests also found significant differences between groups for the change in cortisol (*P* = 0.021) and ACTH (*P* = 0.021) from baseline to treatment endpoint. There were no significant changes within groups or between groups for cortisol, ACTH, GR binding, or lysozyme IC_50-DEX_ at one-month follow-up.

 No side effects, adverse events, or serious adverse events were reported by participants in either treatment condition.

## 4. Discussion

This is the first study to examine mifepristone in PTSD. In these preliminary data, mifepristone was significantly more effective than placebo based on the primary outcomes of clinical responder status and PTSD symptom severity. Rates of remission were also higher in the mifepristone group than the placebo group. Interestingly, these beneficial effects were more marked at four-week follow-up than at treatment endpoint, suggesting that the results do not merely reflect an acute change in neurohormonal activity. The precise central mechanism of action of mifepristone is not known.

Indeed, mifepristone induced acute increases in cortisol and ACTH levels, as would be predicted by blockade of negative feedback inhibition. To our knowledge, this is the first study that also demonstrates changes in glucocorticoid receptor number in lymphocytes with mifepristone. Since cytosolic GR declined substantially following mifepristone administration, the data raise the possibility that the acute increase in cortisol levels may have increased binding to cytosolic GR, resulting in greater translocation to cell nuclei and enhanced glucocorticoid signaling. At four-week follow-up, the acute neurohormonal changes had resolved, but beneficial clinical effects were evident. These findings may reflect some form of recalibration of the HPA axis; mifepristone also impairs consolidation of fear memory [11] and may directly or indirectly exert effects on the inflammatory cascade [12], both of which could be therapeutic in PTSD. 

The results of this pilot study are limited by the small sample size and all the male veteran sample. Additionally, half of all study participants were veterans of the Vietnam or post-Vietnam era; these veterans may have had PTSD of a much longer duration when compared to OIF/OEF veterans, which may confound the results of the study. However, this clinical trial had a high retention rate and no adverse events or complaints about study procedures, suggesting that mifepristone treatment is safe, tolerable, and acceptable to veterans with PTSD, as has been found in other populations. The positive findings of this pilot clinical trial provide an important proof of the concept that treatment with mifepristone is a feasible strategy for improving clinical outcomes in veterans with PTSD; accordingly, further study is indicated. Should this neuroendocrine agent yield evidence of efficacy in PTSD, there are many potential uses which could be explored: as single or intermittent monotherapy in patients reluctant to take long-term medications, as an adjunct to pharmacotherapy, psychotherapy, or other standard treatments, or as prophylaxis in acutely traumatized persons.

## 5. Conclusions

Additional funding is needed to conduct a larger, multisite trial of mifepristone in veterans with PTSD to determine if this medication is an effective psychopharmacological treatment option for PTSD.

## Figures and Tables

**Table 1:** Clinical and neuroendocrine outcomes.

			Pre-treatment	Post-treatment		Between Groups Significance	
			Initial	Endpoint	4-week	Initial to endpoint	Initial to 4-week
			Mean (SD)	Mean (SD)	Mean (SD)	*P* value	*P* value
Clinical	CAPS Score	Drug (*n* = 4)	68.75 (10.24)	43.50 (19.49)	44.00 (24.12)	0.425	**0.047** ^ a^
		Placebo (*n* = 4)	68.25 (8.77)	56.50 (31.89)	65.25 (18.86)		
	Intrusive Sx	Drug (*n* = 4)	13.75 (8.06)	8.00 (7.70)	7.50 (9.00)	0.463	0.126
		Placebo (*n* = 4)	14.50 (4.65)	12.00 (10.30)	15.00 (9.42)		
	Avoidance Sx	Drug (*n* = 4)	29.25 (7.14)	18.75 (11.44)	17.00 (10.74)	0.239	**0.027** ^ b^
		Placebo (*n* = 4)	28.50 (1.29)	25.50 (7.05)	26.00 (5.72)		
	Hyperarousal Sx	Drug (*n* = 4)	25.75 (0.50)	16.75 (5.56)	19.50 (9.68)	0.786	0.359
		Placebo (*n* = 4)	25.25 (6.40)	18.25 (10.53)	24.25 (5.97)		
	BDI	Drug (*n* = 4)	22.25 (5.85)	13.25 (4.72)	11.50 (9.15)	0.124	0.769
		Placebo (*n* = 4)	24.25 (12.12)	24.75 (10.05)	23.75 (9.54)		
	PCL	Drug (*n* = 4)	60 (10.83)	46.75 (11.79)	49.25 (15.59)	0.691	0.200
		Placebo (*n* = 4)	63.25 (11.41)	54.25 (24.17)	56.25 (16.68)		

Neuroendocrine	Cortisol (*μ*g/dL)	Drug (*n* = 4)	13.15 (3.52)	31.28 (5.49)	11.53 (4.27)	**0.001** ^ c^	0.786
		Placebo (*n* = 4)	12.43 (6.16)	12.90 (4.87)	11.83 (2.91)		
	ACTH (pg/mL)	Drug (*n* = 4)	49.33 (29.69)	153.45 (40.25)	53.63 (8.08)	**0.009** ^ d^	0.785
		Placebo (*n* = 4)	30.88 (14.83)	38.40 (16.18)	41.63 (19.33)		
	Lysozyme IC50 (nM)	Drug (*n* = 2)	8.00 (2.55)	5.57 (.76)	6.73 (3.66)	0.162	0.411
		Placebo (*n* = 4)	3.27 (2.89)	3.65 (2.06)	5.03 (1.68)		
	GR/cell	Drug (*n* = 3)	1557.67 (539.75)	188.67 (116.89)	1593.00 (770.02)	**0.003**	0.322
		Placebo (*n* = 4)	1019.50 (553.91)	1243.00 (543.84)	1824.75 (683.80)		

^a^Median change in CAPS score for mifepristone group (20.0); median change in CAPS score for placebo group (10.5); nonparametric *P* value (0.021).

^b^Median change in CAPS avoidance symptom score for mifepristone group (11.0); median change in CAPS avoidance symptom score for placebo group (4.0); nonparametric *P* value (0.028).

^c^Median change in cortisol for mifepristone group (17.2); median change in cortisol for placebo group (0.4); nonparametric *P* value (0.021).

^d^Median change in ACTH for mifepristone group (90.6); median change ACTH for placebo group (4.2); nonparametric *P* value (0.021).
